# Temporal dynamics of TNF-mediated changes in hematopoietic stem cell function and recovery

**DOI:** 10.1016/j.isci.2023.106341

**Published:** 2023-03-05

**Authors:** Alexandra Rundberg Nilsson, Isabel Hidalgo, David Bryder, Cornelis Jan Pronk

**Affiliations:** 1 Medical Faculty, Division of Molecular Hematology, Institution for Laboratory Medicine, Lund University, 22184 Lund, Sweden; 2 Medical Faculty, Lund Stem Cell Center, Lund University, 22184 Lund, Sweden; 3 Medical Faculty, Division of Molecular Medicine and Gene Therapy, Institution for Laboratory Medicine, Lund University, 22184 Lund, Sweden; 4 Wallenberg Center for Molecular Medicine, Lund University, 22184 Lund, Sweden; 5 Childhood Cancer Centre, Skåne University Hospital, 22185 Lund, Sweden

**Keywords:** Biological sciences, Cell biology, Molecular biology, Stem cells research

## Abstract

While tumor necrosis factor (TNF) is a critical mediator of appropriate immune response and tissue repair, its misregulation is linked to cancer, autoimmunity, bone marrow failure, and aging. Understanding the context-dependent roles of TNF is essential for elucidating normal and pathogenic conditions and to guide clinical therapy advancements. Prior studies suggested that TNF restricts the self-renewal capacity of hematopoietic stem cells (HSCs), but its long-term effect on HSCs remains unclear. Here, we demonstrate that *in vivo* TNF administration results in a transient exit of HSCs from quiescence, which coincides with a compromised repopulation capacity. These functional changes are; however, fully reversible even following prolonged/chronic transient exposure to TNF. Notably, antagonizing TNF signaling in transplantation recipients enhances donor HSC reconstitution. Our findings provide molecular and functional insight into HSC regulation, with implications for both acute and chronic inflammatory conditions.

## Highlights

• Temporary TNF exposure results in reversible HSC repopulation capacity reductions
• TNF-induced repopulation impairments coincide with altered quiescence status
• TNF signaling inhibition enhances HSC reconstitution in transplantation recipients

## Graphical abstract

**Figure:**
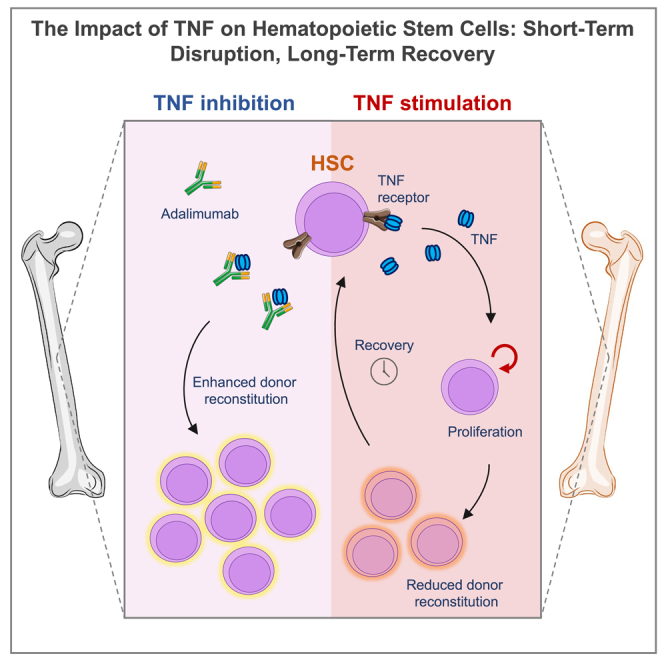

## Introduction

While inflammatory signaling is crucial for hematopoietic development, to mount appropriate immune responses to infections and to promote tissue regeneration, it can also be linked to chronic inflammatory conditions, aging, bone marrow (BM) failure, and cancer.^1^^,^^2^^,^^3^^,^^4^^,^^5^^,^^6^ Hematopoietic stem cells (HSCs) reside at the top of the hematopoietic hierarchy and are ultimately responsible for sustaining stable multilineage hematopoiesis.^7^^,^^8^ While most HSCs remain quiescent during steady-state, infections and injuries offset the balance of HSC dormancy, self-renewal, and differentiation to accommodate the requirements for new blood cells.^9^

The inflammatory mediators tumor necrosis factor (TNF),^10^^,^^11^ type I interferons (IFNs),^12^^,^^13^ IFN-γ^14^, and interleukin (IL)-1^15^ can all induce HSC activation and differentiation. Transplantation experiments using HSCs from subjects acutely exposed to such factors have generally revealed a compromised reconstitution capacity,^10^^,^^14^^,^^16^ although some controversy exists.^17^ As acute exposure to different types of inflammatory mediators leads to similar functional outcomes, a perhaps reasonable assumption has been that chronic exposure would lead to comparable responses. In agreement with this, sustained toll-like receptor (TLR)-4 activation by stimulation with lipopolysaccharide (LPS) resulted in impaired HSC function.^18^^,^^19^ Similarly, chronic polyinosinic:polycytidylic acid (pI:pC) stimulation reduced the repopulating capacity of HSCs, albeit without exhaustion of HSCs in the primary hosts.^12^^,^^13^ By contrast, the impaired HSC repopulation ability after chronic stimulation with IL-1 could be fully restored upon removal of the stimulus and a recuperation period,^15^ suggesting a more adaptable recovery of HSCs to at least some proinflammatory cytokines. A recent study further advanced the aforementioned findings on IL-1 with the demonstration that IL-1 receptor knock-out (KO) mice or pharmacological IL-1 blockage could ameliorate certain HSC aging-phenotypes driven by inflammation.^20^ These results suggest that targeting inflammatory mediators could reverse or mitigate an impaired HSC function in at least a subset of chronic inflammatory conditions.

TNF levels are elevated in several inflammatory conditions, including inflammatory bowel disease,^21^ rheumatoid arthritis,^22^ psoriasis,^23^ aging,^24^ cancer,^25^^,^^26^^,^^27^ and COVID-19.^28^ TNF is also involved in conditions characterized by disrupted hematopoiesis and that drive BM failure syndromes.^3^^,^^4^^,^^5^ Anti-TNF treatment is today’s standard of care for a range of inflammatory conditions and has revolutionized the clinical outcome of these patients.^28^^,^^29^^,^^30^ Despite its clinical success, the mechanisms of action of TNF are multifaceted. For instance, in cancer, TNF can have both anti-tumor and pro-tumorigenic effects.^25^ The influence of TNF on HSCs is also complex. While discrepancies exist regarding both positive or negative effects on survival and proliferation, recent studies have suggested distinct responses by HSCs and other more committed progenitors, such as granulocyte-macrophage progenitors (GMPs).^10^ This divergence may, at least partly, have influenced the interpretations from experiments in which HSCs were exposed to continuous TNF while differentiating through more apoptosis-prone progenitor states. In addition to such limitations, *in vitro* conditions cannot fully recapitulate the complex environment of cells and factors that apply to more physiologically relevant *in vivo* settings. Furthermore, TNF signals through two distinct receptors, TNFR-p55 and TNFR-p75 that diverge in their affinities to soluble and membrane-bound TNF and their mediated signaling transduction.^31^ With the objective to unravel mechanisms relevant for acute and chronic clinical settings, we here focused on using *in vivo* assays to assess the impact of transient short and prolonged TNF exposure on HSCs. We focused primarily on the capacity of HSCs to recuperate functional properties following the disruption of TNF exposure, a notion of direct clinical relevance in patients suffering from diseases involving TNF.

## Results

### Acute TNF exposure forces quiescent HSCs into an active cell cycle and depletes lineage-committed hematopoietic cells

**Figure 1:**
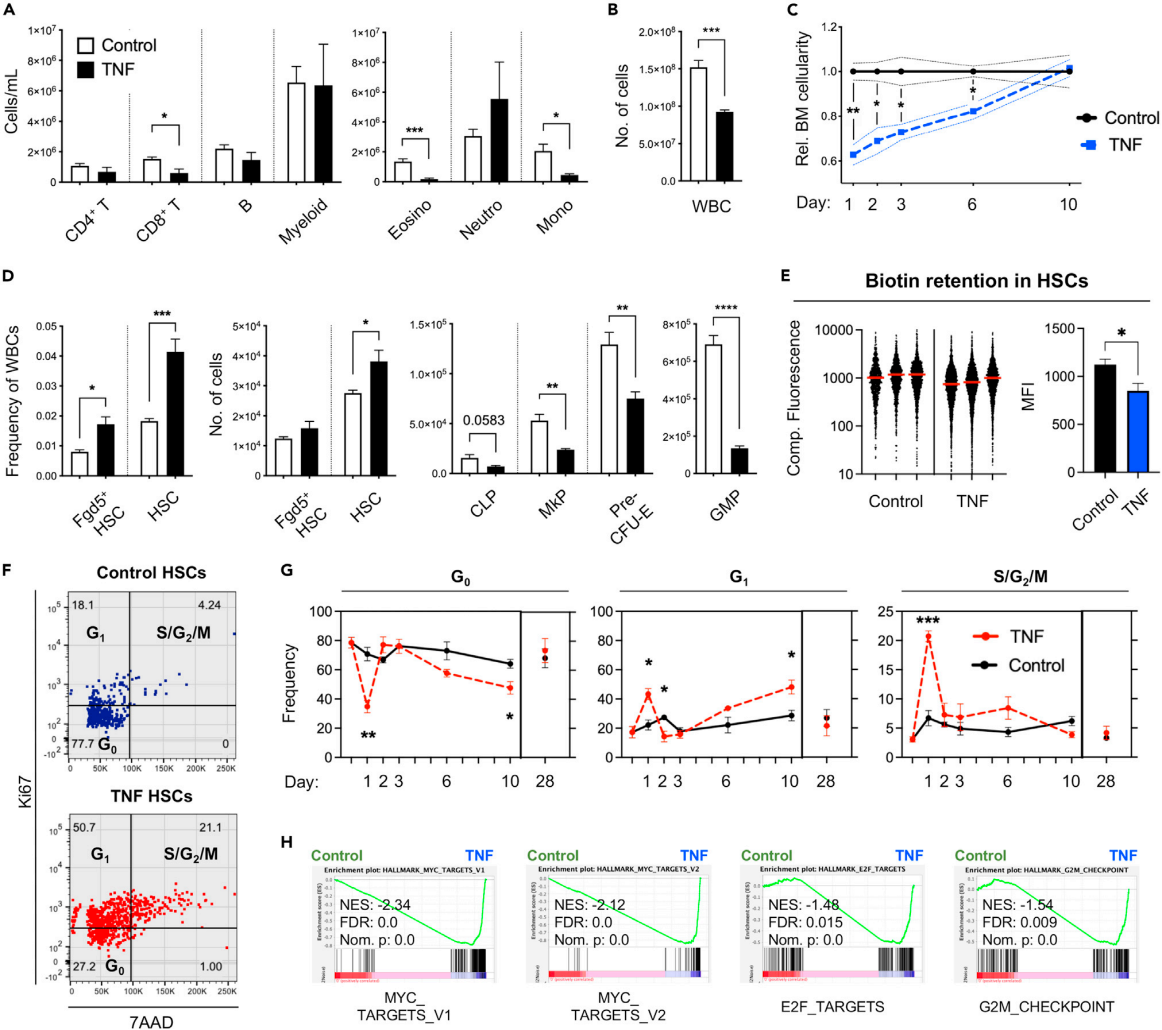
Acute TNF exposure transiently decreases bone marrow cellularity and lineage-committed progenitors while increasing HSC proliferation and self-renewal (A) PB cell concentrations of mature blood cell subsets. (B) BM white blood cell (WBC) cellularity. (C) BM WBC cellularity over time after TNF administration. Control n = 3, TNF n = 3 for all time points. (D) HSC frequencies and cell numbers, and numbers of defined lineage-committed progenitor cells in the BM one day post TNF administration. Eosino = eosinophils, neutro = neutrophils, mono = monocytes. TNF-treated mice n = 5, control-treated mice n = 4. (E) Proliferation history by biotin retention in HSCs two days post TNF administration. (Left) Compensated fluorescence distribution of biotin in HSCs. Red line represents mean. Control samples: 997, 1,187, and 1,709 events, TNF samples: 3,293, 4,228, and 2,262 events. 1,362 data points are outside the limits of the axis. (Right) Quantification of biotin data: mean fluorescence intensity (MFI) for each sample. Control n = 3, TNF n = 3. (F) Representative cell cycle distribution plots for HSCs at one day post TNF exposure. (G) HSC cell cycle distribution at various time points after TNF administration. Control n = 3, TNF n = 3 for all time points. (H) Gene set enrichment analysis (GSEA) of proliferation-associated gene sets in HSCs 3 h post exposure to TNF. Error bars in A, B, D, and E represent +SEM. Dotted lines in C and error bars in G represent ±SEM. Comparisons were done using unpaired two-tailed student’s t-tests, ∗p < 0.05, ∗∗p < 0.01, ∗∗∗p < 0.001, ∗∗∗∗p < 0.0001.

We began to assess the acute hematopoietic responses following *in vivo* TNF administration. For this, we intravenously injected mice with a single dose of TNF (2 μg/mouse). On the following day, we monitored the cellularity of different blood cell types in peripheral blood (PB) (Figure 1A) and of phenotypically defined hematopoietic stem and progenitor cells (HSPCs) in the BM (Figure 1D).

In PB, TNF had only minor effects on overall white blood cell (WBC) cellularity, with a stronger effect on platelet (PLT) counts (Figure S1A). Investigating this in more detail by flow cytometry (Figure S2A) revealed significantly reduced numbers of CD8^+^ T cells (2.5-fold) and a distinct redistribution within the myeloid cell compartment. While eosinophils and monocytes drastically declined, the numbers of neutrophils increased (Figure 1A), thereby corroborating the role of TNF in neutrophil recruitment following inflammation.^32^^,^^33^

In the BM, and in agreement with previous studies,^10^^,^^11^ we observed a more prominent effect of TNF on overall cellularity (Figure 1B). Monitoring this effect over time revealed an essentially linear recovery with cell counts returning to baseline levels 10 days after administration (Figure 1C). Despite these acute effects, we observed that both the frequency and the absolute numbers of HSCs (as defined by a Lin^−^Sca^+^Kit^+^CD48^−^CD150^+^ phenotype, Figure S2B) were increased following TNF treatment (Figure 1D). As inflammation can alter the phenotype of HSCs,^13^^,^^18^ we assessed the acute TNF effects on HSCs using an Fgd5-ZsGreen transgenic reporter mouse. Fgd5 expression is nearly exclusive to stringently purified HSCs^34^ (Figure S2B) and was previously shown to be unaffected by inflammation.^35^ The results using Fgd5^+^ HSCs confirmed that TNF did not negatively influence on HSC numbers and, if anything, rather associated with their slight expansion (Figure 1D). In contrast to the effects on HSCs, *in vivo* TNF stimulation led to a pronounced attenuation of early lineage-committed progenitors, including for common lymphoid progenitors (CLPs, 2.1-fold reduction), granulocyte-macrophage progenitors (GMPs, 5.1-fold reduction), megakaryocyte progenitors (MkPs, 2.2-fold reduction), and erythroid progenitors (Pre-CFU-Es, 1.7-fold reduction) (Figures 1D and S2C). While the remaining cells within the Lin^−^Sca^+^Kit^+^ (LSK) fraction, which reside developmentally in between HSCs and lineage-committed progenitors, were unaltered, other myelo-erythroid progenitors were also reduced (Figure S1B).

To further our investigations on TNF-mediated HSC responses, we assessed the proliferation status of HSCs up to 28 days after TNF administration. This revealed that HSCs subjected to TNF were significantly less quiescent one day after treatment, with a substantially larger portion of cells in both G_1_ and S/G_2_/M phases (Figures 1F, 1G, and S1C). These results were complemented with results from an *in vivo* biotin retention assay,^36^ which revealed that more HSC divisions had occurred two days following exogenously delivered TNF (Figure 1E). The strong initial increase in HSC proliferation was followed by a milder activation from day 6–10 that was mainly associated with an increased fraction of HSCs in G_1_ (Figure 1G). Importantly, no differences in HSCs cell cycle activity could be observed between the groups 28 days post TNF administration.

To determine if we could identify transcriptional underpinnings to our observed cell cycle activation, we took advantage of a previously generated RNA-sequencing dataset (GSE115403).^10^ In this, triplicate biological samples of HSCs (defined phenotypically according to same criteria as we used in this work) were isolated from mice 3 h after single injections of TNF or PBS and thereafter subjected to RNA sequencing. We approached these data by gene set enrichment analyses (GSEA) using the proliferation-associated molecular signature database (MSigDB) hallmarks gene sets for MYC targets, E2F targets, and G_2_M checkpoint.^37^ In support of the observed changes in cell cycle distribution (Figures 1F and 1G), *in vivo* TNF stimulation resulted in a striking enrichment of the proliferation-associated pathways (Figure 1H).

Taken together, these results demonstrated that the acute effects of TNF on hematopoiesis included depletion of lineage-committed progenitors and driving HSCs out from quiescence in a rapid and transient manner.

### TNF mediates transient functional impairments to hematopoietic stem cells

**Figure 2:**
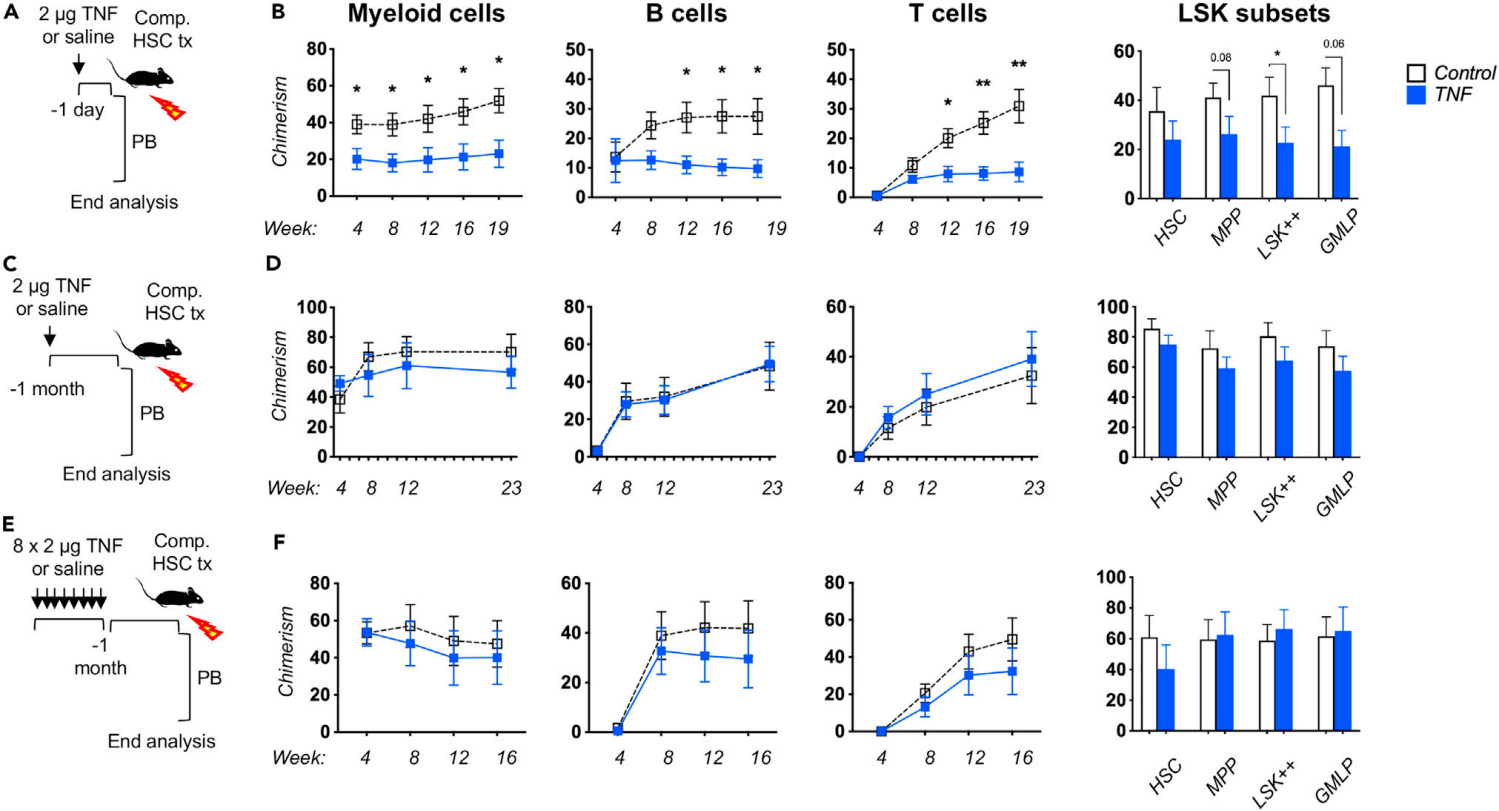
Acute and chronic TNF exposure transiently reduces HSC repopulation capacity (A) Experimental strategy to assess the activity of HSCs receiving one dose of TNF or saline control one day prior to transplantation. The results are depicted in Figure 2B. (B) PB myeloid cell, B cell, and T cell chimerism over time after transplantation, as well as BM LSK subset chimerism levels in recipient mice 19 weeks post transplantation. Recipients: WT n = 9, TNF n = 8. (C) Experimental strategy to assess the activity of HSCs receiving one dose of TNF or saline control one month prior to transplantation. The results are depicted in Figure 2D. (D) PB myeloid cell, B cell, and T cell chimerism over time after transplantation, as well as BM LSK subset chimerism levels in recipient mice 23 weeks after transplantation. WT n = 6, TNF n = 5. (E) Experimental strategy to assess the activity of HSCs receiving 8 doses of TNF or saline control one month prior to transplantation. The results are depicted in Figure 2F. (F) PB myeloid cell, B cell, and T cell chimerism over time after transplantation, as well as BM LSK subset chimerism levels in recipient mice 16 weeks after transplantation. WT n = 6, TNF n = 5. For all three settings, 100 HSCs were transplanted together with 300,000 unfractionated BM competitor cells into lethally irradiated recipients. Error bars represent ±SEM. Comparisons were done using unpaired two-tailed student’s t-tests, ∗p < 0.05, ∗∗p < 0.01, ∗∗∗p < 0.001, ∗∗∗∗p < 0.0001.

We next compared the functional consequences of acute and chronic TNF exposure on HSC function. For this, we injected mice with a single dose of TNF or saline control, followed by HSC extraction and competitive transplantation one day (Figures 2A and 2B) or one month (Figures 2C and 2D) after treatment. In a third group, we administered TNF or saline control to donor mice for three weeks (8 consecutive injections with 3-day intervals) to approximate chronic TNF activation. After this treatment regimen, mice were again allowed to recover for a month before HSC extraction and transplantation (Figures 2E and 2F). In both these latter settings, HSCs had returned to quiescence by the time of extraction (data not shown). Transplanted mice were subsequently analyzed by assessing donor HSC-derived chimerism in PB up to 23 weeks post transplantation, after which HSPCs in the BM were also analyzed.

When HSCs were transplanted one day after TNF treatment, we observed a significantly reduced reconstitution capacity that affected all evaluated lineages similarly, and that extended to immature HSPCs in the BM (Figure 2B). This was confirmed in competitive transplantation experiments using Fgd5^+^ HSCs (Figures S3A–S3C). In addition to the previously mentioned functional and transcriptional increase in HSC cycle activity that we observed following TNF administration, gene expression analysis revealed dysregulation of several pathways and genes in support of a reduced HSC reconstitution potential (Table 1). For instance, downregulated genes (Table S1) enriched for the Rho GTPase cycle, including *Rac2* (Table S2), which has been linked to HSC retention, engraftment, and survival.^38^ Moreover, pathways linked to an active HSC state with reduced reconstitution capacity as opposed to more dormant or quiescence HSCs, such as gene expression, protein processing, and translation, as well as unfolded protein response (UPR) were upregulated (Tables S3 and S4). Furthermore, apoptosis and IFN-gamma signaling^39^, both linked to reduced HSC reconstitution capacity, were enriched among upregulated genes.

**Table 1:** TNF induces differential regulation of key cellular pathways in HSCs with implications for survival, reconstitution, and differentiation

	Adj. p value	Combined score	+TNF	Consequence
**BioPlanet**				

Rho GTPase cycle	0.008	65.07	Down	Expression is linked to HSC retention, engraftment, and survival.
Protein processing in the endoplasmic reticulum	1.68e-14	347.25	Up	Low levels of transcription and translation are linked to undifferentiation and dormancy.
Gene expression	7.31e-9	67.59	Up	
Protein metabolism	3.90e-7	69.26	Up	

**MSigDB Hallmark**				

Interferon gamma response	5.964e-14	239.19	Up	Increased signaling is associated with apoptosis and reduced reconstitution capacity.
Unfolded Protein Response	4.504e-9	162.62	Up	Responds to accumulation of unfolded/misfolded proteins. Controls cell viability and function.
Apoptosis	0.0002246	36.58	Up	Cell death.

Enrichment of HSC potency/reconstitution-associated gene sets upon HSC TNF exposure.

The observation from the HSCs transplanted one day after TNF administration was in striking contrast to HSCs isolated from mice that were allowed to recover for a month after the treatment. In the recipients of these cells, we failed to observe any differences between control and TNF treated HSCs neither short- nor long-term in PB, nor in chimerism levels of BM HSPCs at the experimental endpoint (Figure 2D). Finally, when assessing the effects of more extended TNF exposure, but again allowed to recover for a month before functional assessments, we similarly did not observe any functional consequences of TNF exposure on HSC function (Figure 2F).

Taken together, these results demonstrated that the impairments mediated by TNF on HSC function were restricted to the immediate period during which the quiescence of HSCs is relaxed. Importantly, this relaxation did not associate with any signs of functional HSC exhaustion or long-term alterations, neither upon limited nor more chronic transient TNF exposure.

### TNF inhibition improves donor reconstitution in a direct manner

TNF levels are increased in the BM environment following irradiation,^40^ which is commonly used for myeloablative conditioning prior to hematopoietic cell transplantation. Given the negative effects that have been linked to TNF exposure, we were interested to know whether recipient-administered TNF inhibition in connection to hematopoietic transplantation could positively influence on WT HSC reconstitution activity in this setting where TNF levels are endogenously elevated without the addition of exogenous TNF. Importantly, as several TNF inhibitors are available this is a clinically tractable procedure.

**Figure 3:**
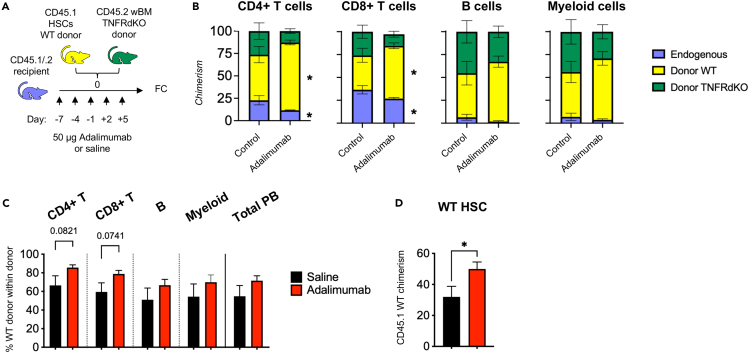
Antagonization of recipient TNF signaling increases donor reconstitution (A) Experimental strategy to assess the consequences of recipient TNF-inhibitory treatment on transplantation outcome. WT CD45.1/.2 double-positive recipients were injected i.p with 50 μg of the TNF inhibitor adalimumab between 7 days prior to 5 days post transplantation in 3-day intervals. 100 CD45.1 WT HSCs were transplanted together with 300,000 unfractionated CD45.2 TNFR-dKO competitive BM cells. Results are depicted in B-D. (B) PB donor chimerism of endogenous (double-positive CD45.1+/2+), donor WT (CD45.1+), and donor TNFR-dKO (CD45.2+) cells 18 weeks post transplantation. Error bars represent ±SEM. (C) PB CD45.1 WT chimerism within donor-WBCs (CD45.1+ and CD45.2+, excluding recipient double-positive CD45.1+/.2 + cells) at 18 weeks post transplantation. (D) Donor WT HSC chimerism in the BM 18 weeks post transplantation. Error bars in C and D represent +SEM. Control n = 6, adalimumab n = 7. Comparisons were done using unpaired two-tailed student’s t-tests, ∗p < 0.05, ∗∗p < 0.01, ∗∗∗p < 0.001, ∗∗∗∗p < 0.0001.

We began by treating F1 CD45.1 x CD45.2 WT recipient mice with the TNF inhibitor adalimumab (or saline to control recipients) every third day from seven days prior to irradiation/transplantation until five days post transplantation (a total of 5 intraperitoneal injections; Figure 3A). Following irradiation of these mice when TNF levels normally rise, they were transplanted with WT HSCs (CD45.1) and unfractionated competitor BM cells from TNFR-dKO mice (CD45.2), which lack both TNF receptors and should thus be unaffected by direct TNF stimulation. This setup allowed us to assess the contribution from donor WT HSCs in the presence or absence of adalimumab against the treatment-indifferent TNFR-dKO reference, and at the same time distinguish endogenous from donor-derived hematopoiesis. We observed that adalimumab-treated mice had significantly lower endogenous contribution and significantly higher donor WT contribution to PB T cell compartments 18 weeks post-transplantation (Figure 3B). This was paralleled by a higher WT T cell chimerism within the donor compartment (Figure 3C), as well as significantly increased WT donor HSC chimerism in the BM (Figure 3D). The endogenous B and myeloid cells, which have considerably higher peripheral turnover than those of T cells and are not known to survive to a significant extent upon lethal irradiation were virtually absent in both groups, and a potential difference in the endogenous response for these compartments could therefore, not be assessed. While the WT donor chimerism showed large variations in both groups, a tendency to increased chimerism in the adalimumab groups could be observed in all investigated compartments.

Taken together, these results demonstrated that inhibition of TNF in connection to conditioning can improve the reconstituting capacity of transplanted HSCs.

## Discussion

In this work, we closely examined the consequences of TNF signaling on HSC performance. Importantly, we could demonstrate that TNF withdrawal led to a full reversal of the adverse effects on HSC repopulating activities following both acute and chronic TNF stimulation. This mimics the situation in which a transiently reduced BM function is associated with infections that are characterized by increased TNF levels.^41^

HSC recuperation has previously been observed after removal of chronic IL-1β exposure,^15^ and anti-IL-1 treatment can ameliorate several age-related changes to HSCs.^20^ These results suggest that TNF and IL-1β modulate long-term HSC capacity through reversible mechanisms, as opposed to the irreversible HSC changes that are induced by chronic stimulation with LPS^18^^,^^19^ and pI:pC.^12^^,^^13^ Inflammatory signaling is; however, intricate and their mediators including TNF, IL-1β, IL-6, and interferons often co-occur in conditions such as aging,^42^ cancer,^26^^,^^27^ and autoimmunity.^43^ *In vivo* administration of LPS has previously shown to increase *Il6* and *Il1b* but not *Tnfa* transcripts in HSCs,^19^ but whose proteins were only secreted from other cells within the LSK compartment upon *in vitro* LPS treatment.^44^ Furthermore, we observed that TNF induced IFN-α, IFN-γ, and IL-6 signaling in HSCs (data not shown). This complexity suggests that not solely the presence, but also the order, levels, duration, and/or sources of interacting inflammatory mediators can determine the long-term cellular consequences on HSCs. While further studies are required to decipher the exact mechanisms operational in HSCs, we believe that our findings have potential consequences for the understanding and treatment of a plethora of clinical conditions that present with TNF dysregulation.

TNF has previously shown to induce HSC proliferation.^10^ We confirmed this in our current study and could additionally, identify the transcriptional underpinnings of this enforced cell cycle activation. We could also demonstrate that *in vivo* stimulation with TNF resulted in increased numbers and self-renewal of phenotypical HSCs, concomitant with a vast reduction of all evaluated lineage-committed progenitors one day following a single dose of TNF. Of note, the relative contribution of cell death, proliferation, and differentiation was not established, and it appears likely that all these processes could be involved. Nevertheless, HSCs appeared to be uniquely protected from TNF-mediated depletion in the BM.

TNF increases in response to irradiation, a procedure that is frequently utilized for lympho- and myeloablation in the preparation for HSC transplantation in both clinical and research settings. It has been previously suggested that irradiation-induced TNF causes production of reactive oxygen species (ROS) and that treatment with the antioxidant *N*-acetyl-L-cysteine (NAC) prior to transplantation could therefore increase HSC reconstitution.^40^ Several studies have similarly focused on achieving increased engraftment by various treatments applied to the donor cells.^45^ Although successful improvements have occasionally been achieved,^40^^,^^46^ the culture conditions used in such studies are regardless of treatment often associated with HSC differentiation and reduced HSC reconstitution capacity compared to non-cultured cells.^47^ Here, we showed that *in vivo* administration of the clinically widely used TNF inhibitor adalimumab to irradiated transplantation recipients increased the donor contribution from transplanted HSCs. Although the endogenous myeloid and B cell contribution was virtually absent in both groups and the consequences to these cells could therefore not be assessed, the endogenous T cell contribution was significantly reduced in the adalimumab-treated group. This finding could potentially be advantageous for reducing graft rejection, although allograft situations also exist where the presence of TNF might be beneficial for reconstitution and induction of transplantation tolerance.^48^ Importantly, while anti-TNF treatment decreased the contribution from endogenous WT cells, the contribution from donor WT cells increased.

Collectively, our findings clarify and elaborate on our understanding of the regulatory roles of TNF on HSCs. We have shown that HSCs rapidly respond to *in vivo*-administered TNF by altering their proliferation and repopulation activity. The transient exit of HSCs from quiescence that follows TNF exposure is accompanied by an impaired repopulation capacity that is fully restored upon return to quiescence, even after chronic TNF exposure. This notion is reassuring, given the central role of TNF upregulation in many infections and (auto-)inflammatory diseases. It also holds potential for the advancement of clinical therapies aimed at increasing donor reconstitution following transplantation.

### Limitations of the study

One limitation with our study is that the levels of TNF exposure to HSCs may be higher than what is physiologically relevant. However, the optimal level of exogenously administered TNF required to achieve physiologically relevant levels in the BM, and how this relates to human physiology, remain unclear. In this study, we intravenously injected 2 μg of TNF to mice, a concentration that allows for comparison with previous key studies.^10^^,^^11^ In light of this, our findings demonstrate an even more impressive resilience of HSCs to recover from TNF exposure and provide valuable insights into the regulatory role of TNF in HSC function.

## STAR★Methods

### Key resources table

**Table:** 

REAGENT or RESOURCE	SOURCE	IDENTIFIER
**Antibodies**		

Biotin anti-mouse/human CD45R/B220 (RA3-6B2)	BioLegend	Cat: 103204; RRID:AB_312989
Biotin anti-mouse CD4 (GK1.5)	BioLegend	Cat: 100404; RRID:AB_312689
Biotin anti-mouse CD8a (53-6.7)	BioLegend	Cat: 100704; RRID:AB_312743
Biotin anti-mouse/human CD11b (M1/70)	BioLegend	Cat: 101204; RRID:AB_312787
Biotin anti-mouse Ly-6G/Ly6C (Gr-1) (RB6-8C5)	BioLegend	Cat: 108404; RRID:AB_313369
Biotin anti-mouse TER-119/Erythroid Cells (TER-119)	BioLegend	Cat: 116204; RRID:AB_313705
PE/Cy5 anti-mouse/human CD45R/B220 (RA3-6B2)	BioLegend	Cat: 103209; RRID:AB_312994
PE/Cy5 anti-mouse/human CD11b (M1/70)	BioLegend	Cat: 101209; RRID:AB_312792
PE/Cy5 anti-mouse Ly-6G/Ly6C (Gr-1) (RB6-8C5)	SONY	Part: 1142050
PE/Cy5 anti-mouse TER-119/Erythroid Cells (TER-119)	BioLegend	Cat: 116209; RRID:AB_313710
PE/Cy5 anti-mouse CD3*ε* (145-211)	BioLegend	Cat: 100309; RRID:AB_312674
APC/Cy7 anti-mouse CD4 (RM4-5)	BioLegend	Cat: 100526; RRID:AB_312727
PE/Cy5 anti-mouse CD8a (53-6.7)	BioLegend	Cat: 100709; RRID:AB_312748
APC anti-mouse human CD11b (M1/70)	BioLegend	Cat: 101212; RRID:AB_312795
Al700 anti-mouse CD3 (17A2)	SONY	Part: 1101080
PE/Cy7 anti-mouse CD19 (6D5)	BioLegend	Cat: 115520; RRID:AB_313655
PE anti-mouse CD45.1 (A20)	BioLegend	Cat: 110708; RRID:AB_313497
FITC anti-mouse CD45.2 (104)	BioLegend	Cat: 109806; RRID:AB_313443
APC-eFluor780 anti-mouse CD117 (c-Kit) (2B8)	Invitrogen	Ref: 47-1171-82; RRID:AB_1272177
Pacific Blue anti-mouse Ly-6A/E (Sca-1) (E13-16.7)	BioLegend	Cat: 122520; RRID:AB_2143237
Alexa Fluor 700 anti-mouse CD48 (HM48-1)	BioLegend	Cat: 103426; RRID:AB_10612755
PE/Cy7 anti-mouse CD48 (HM48-1)	BioLegend	Cat: 103424; RRID:AB_2075049
APC anti-mouse CD150 (SLAM) (TC15-12F12.2)	BioLegend	Cat: 115910; RRID:AB_493460
PE anti-mouse CD135 (FLT3) (A2F10))	BioLegend	Cat: 135306; RRID:AB_1877217
PE/Cy7 anti-mouse CD105 (MJ7/18)	BioLegend	Cat: 120410; RRID:AB_1027700
PerCP-eFluor710 anti-mouse CD41 (eBioMWReg30)	Invitrogen	Ref: 46-0411-82; RRID:AB_10855042
PE anti-mouse CD41 (MWReg30)	BioLegend	Cat: 133906; RRID:AB_2129745
Alexa Fluor 700 anti-mouse CD16/32 (93)	Invitrogen	Ref: 56-0161-82; RRID:AB_493994
FITC anti-mouse Ki67	BD Biosciences	Cat: 556026; RRID:AB_396302
PE anti-mouse Ki67	BD Biosciences	Cat: 556027; RRID:AB_2266296
Brilliant Violet 605 Streptavidin	BioLegend	Cat: 405229

**Chemicals, peptides, and recombinant proteins**		

Recombinant murine TNF-*α*	PeproTech	Cat: 315-01A
EZ-Link® Sulfo-NHS-LC-LC-Biotin	Thermo Scientific	Cat: 21338
7-Aminoactinomycin D (7-AAD)	Invitrogen	Cat: A1310
Propidium Iodide	Molecular Probes	Cat: P3566
Adalimumab (TNF*α* inhibitor)	Abbvie	ATC: L04AB04

**Critical commercial assays**		

BD cytofix/cytoperm fixation/permeabilization solution kit	BD Bioscience	Cat: BD 554714

**Experimental models: Organisms/strains**		

Mouse: C57Bl/6JRj	Janvier Labs	Strain name: C57BL/6JRj
Mouse: B6.SJL (B6.SJL-Ptprc^a^/BoyAiTac)	In-house breeding	Taconic Bioscience, Model number 4007
Mouse: Tnfrsf1-dKO	In-house breeding	Jackson Laboratory, stock number 003243

**Software and algorithms**		

FlowJo	BD, Treestar	v. 10
GraphPad Prism	Dotmatics	v. 9
Gene Set Enrichment Analysis (GSEA) software	Broad institute	http://software.broadinstitute.org/gsea/index.jsp
Qlucore Omics Explorer	Qlucore AB	https://qlucore.com/omics-explorer
Enrichr	Chen, E.Y., et al. (2013)^49^. https://doi.org/10.1186/1471-2105-14-128; Kuleshov, M.V. et al. (2016)^50^. https://doi.org/10.1093/nar/gkw377; Xie, Z. et al. (2021). https://doi.org/10.1002/cpz1.90	https://maayanlab.cloud/Enrichr/

**Other**		

RNA-seq. data	Yamashita, M. and Passegue, E. (2019)^10^https://doi.org/10.1016/j.stem.2019.05.019	GEO: GSE115403, samples GSM3177653, GSM3177654, GSM3177655, GSM3177656, GSM3177657, and GSM3177658.

### Resource availability

#### Lead contact

Further information and requests for resources and reagents should be directed to the lead contact, Alexandra Rundberg Nilsson (alexandra.rundberg_nilsson@med.lu.se).

#### Materials availability

This study did not generate new unique reagents.

### Experimental model and subject details

#### Mice

8-14 week old female wild-type C57BL/6 and B6.SJL mice were used throughout the study with the following exceptions: 17-18 week old females were used for biotin experiments. 6 month old male Tnfrsf1-dKO mice^11^ were used as competitive bone marrow. Mice were maintained at the Lund University Biomedical Center vivarium. All procedures were conducted in accordance with ethical permits obtained from the Malmö/Lund animal ethics board (18055/2020 and M186-15).

### Method details

#### Treatments

##### TNF administration

Age- and gender-matched mice were administered 2 μg of recombinant murine TNF (PeproTech, Inc.) intravenously. Controls were administered a volume-equivalent 0.9% saline solution.

##### Adalimumab administration

Adalimumab (Humira, Abbvie, Illinois, USA) was administered intraperitoneally at 50 μg/dose, every 3^rd^ day totaling 5 doses per mouse, during a period from 7 days before to 5 days after lethal irradiation and transplantation. Volume-equivalent saline was administered to control mice.

#### Isolation and analysis of peripheral blood and bone marrow

PB and BM cells were isolated and prepared as previously described.^51^^,^^52^ Briefly, PB was collected in EDTA-coated tubes and subjected to cellularity count using Sysmex Hemato Analyzer KX-21N. PB was diluted in FACS buffer (PBS containing 2% FCS and 0.2 mM EDTA), and mixed 1:2 with 2% Dextran in PBS. Samples were subsequently put in a 37°C heating block for 25 minutes after which the upper phase was isolated. FACS buffer was added to the upper phase and samples were centrifugated at 350 g for 5 minutes. Pellets were resuspended in room-temperature lysis solution (STEMCELL Technologies Inc.) and incubated for 2 minutes before washed with FACS buffer, centrifugated, and proceeded to staining with antibodies. Antibodies used for PB cells: biotinylated anti-CD4 APC-Cy7 (clone: RM4-5), anti-CD8 PE-Cy5 (clone: 536.7), anti-CD11b APC (clone: M1/70), anti-CD3 Al700 (clone: 17A2), anti-CD19 PE-Cy7 (clone: 6D5), anti-CD45.1 PE (clone: A20), and anti-CD45.2 FITC (clone: 104). Flow cytometry gating profiles are available in Figure S2A. For BM extraction, tibia, femur and hip bones were collected from each mouse into 4°C FACS buffer. Bones were pestled in a mortar, filtered and counted. After centrifugation, BM cells were lineage-depleted (B220, CD4, CD8, CD11b, Gr-1, and -Ter119) using the MACS magnetic microbead kit (Miltenyi Biotec) according to the manufacturer’s instructions and before subsequent staining. The following antibodies were used for BM staining: biotinylated anti-B220 (clone: RA3-6B2), -CD4 (clone: GK1.5), -CD8 (clone: 53-6.7), -CD11b (clone: M1/70), -Gr-1 (clone: RB6-8C5), and -Ter119 (clone: TER-119), PE/Cy5 conjugated anti-B220 (clone: RA3-6B2), -CD3 (clone: 145-211), -CD8 (clone: 53-6.7), -CD11b (clone: M1/70), -Gr-1 (clone: RB6-8C5), and -Ter119 (clone: TER-119), anti-cKit APC-e780 (clone: 2B8), anti-Sca1 Pacific Blue (clone: E13-161.7), anti-CD48 Al700 and PE-Cy7 (clone: HM48-1), anti-CD150 APC (clone: TC15-12F12.2), anti-FLT3 PE (clone: A2F10), anti-CD45.1 PE, anti-CD45.2 FITC, anti-CD105 PE-Cy7 (clone: MJ7/18), anti-CD41 PerCP-e710 and PE (clone: MWReg30), and anti-CD16/32 Al700 (clone: 93). Flow cytometry gating profiles are available in Figures S2B and S2C. Streptavidin-BV605 was used to identify biotinylated antibodies. 1 μg/mL Propidium Iodide (Molecular Probes) was used to determine viability. Cells were acquired and sorted using FACSAriaIIu, FACSAriaIII, and LSR Fortessa (Becton Dickinson and Company, Franklin Lakes, NJ). Flow cytometry data was analyzed using FlowJo software (Tree Star, Ashland, OR).

#### *In vivo* HSC proliferation analysis

##### Ki67

The cell cycle status of HSCs was determined using the PE and FITC Mouse Anti-Ki-67 Set and the Cytofix/Cytoperm Solution Kit (BD Pharmingen, Becton, Dickinson and Company, Franklin Lakes, NJ) according to the manufacturer’s instructions. Briefly, lineage-depleted BM was stained with antibodies used to define HSCs as described above. After staining, the pelleted cells were fixed and permeabilized by resuspension in BD Fix/Perm™ buffer for 20 minutes at 4°C. Cells were then washed twice with BD Perm/Wash buffer and stained with anti-Ki67 or IgG1 isotype control for 30 minutes, after which cells were washed twice and resuspended in 10 μg/mL 7-Aminoactinomycin D (7-AAD) diluted in Wash/Perm solution over night before analysis.

##### Biotin

HSC proliferation history was analyzed by intravenously injecting mice with 1 mg/4g body weight freshly prepared EZ-Link® Sulfo-NHS-LC-LC-Biotin. 2 days later, the mice were administered 2 μg TNF or saline intravenously and were subsequently euthanized 2 days following the TNF/saline administration to analyze proliferation history by biotin retention. BM cells were stained for HSC markers and streptavidin-BV605 was used to identify the biotin retention.

#### Transplantations

All transplantations were carried out competitively with FACS purified HSCs as previously described.^51^ Briefly, 100 HSCs were sorted and transplanted together with 300 000 unfractionated BM competitor cells into lethally irradiated 8-12 weeks old recipient mice that had been subjected to 800 cGy. Recipient PB was analyzed repeatedly at indicated time-points and BM was analyzed at 16-23 weeks post transplantation.

#### Gene expression analysis

The GSE115403 data set,^10^ which includes the HSC transcriptional profiles from 3 control and 3 TNF-treated mice was used for gene expression analysis. Gene Set Enrichment Analysis (GSEA) was performed as previously described^52^ using the Broad Institute GSEA software.^53^ Enrichment analysis was also performed on significantly up- and downregulated genes. Differentially expressed genes (DEGs) were identified using two group analysis with thresholds of p < 0.05, corrected p value (q value) < 0.1, and fold change >2, using the Qlucore Omics Explorer 3.6 software. With these criteria, we identified 504 and 349 genes were up- and downregulated, respectively, in TNF-exposed HSCs, which were subjected to enrichment analysis using Enrichr.^49^^,^^50^

### Quantification and statistical analysis

Unpaired two-tailed student’s t-tests were applied to calculate statistical significance between groups in flow cytometry experiments using Graphpad Prism. The statistical details can be found in the corresponding figure legends. p-values <0.05 were considered significant. Mice used for treatment and transplantation were randomized into groups and was not blinded. Transplanted mice with donor reconstitution that did not exceed 1% in all evaluated lineages and/or within the HSC pool were considered unsuccessful and excluded from analysis. In accordance with these criteria, the following mice were excluded from analysis: 1) one TNF-treated mouse related to Figures 2A and 2B, 2) one TNF-treated mouse related to Figures 2D that spontaneously succumbed >12 weeks after transplantation, 3) one TNF-treated mouse related to Figures 2F, 4) one saline-treated and one TNF-treated mouse related to Figures S3B and S3C, and 5) one saline-treated mouse related to Figures S3B and S3C. Statistical methods were not used to determine the number of mice required for each experiment.

## Data Availability

• RNA-sequencing data was not generated in this study but was obtained from GEO and is publicly available (NCBI GEO accession number: GSE115403). • No new code was generated in this study. • Any additional information required to reanalyze the data reported in this paper will be shared by the lead contact upon request.

## Supplemental information

Document S1. Figures S1 and S2 and Tables S2 and S4

Table S1. Differentially expressed genes in TNF-exposed compared to WT HSCs, related to Table 1

Table S3. Upregulated pathways in TNF-exposed HSCs identified through gene set enrichment analysis, related to Table 1 BioPlanet gene sets, adj. p-value <0.05.
