# Supplementary material for: Temporal dynamics of TNF-mediated changes in hematopoietic stem cell function and recovery
Source: iScience. 2023 Mar 5;26(4):106341. doi: 10.1016/j.isci.2023.106341 (PMC10031135; doi:10.1016/j.isci.2023.106341)
Supplement: Document S1. Figures S1 and S2 and Tables S2 and S4 [file mmc1.pdf]

iScience, Volume *26*

Supplemental information

Temporal dynamics of TNF-mediated changes in hematopoietic stem cell function and recovery

Alexandra Rundberg Nilsson, Isabel Hidalgo, David Bryder, and Cornelis Jan Pronk

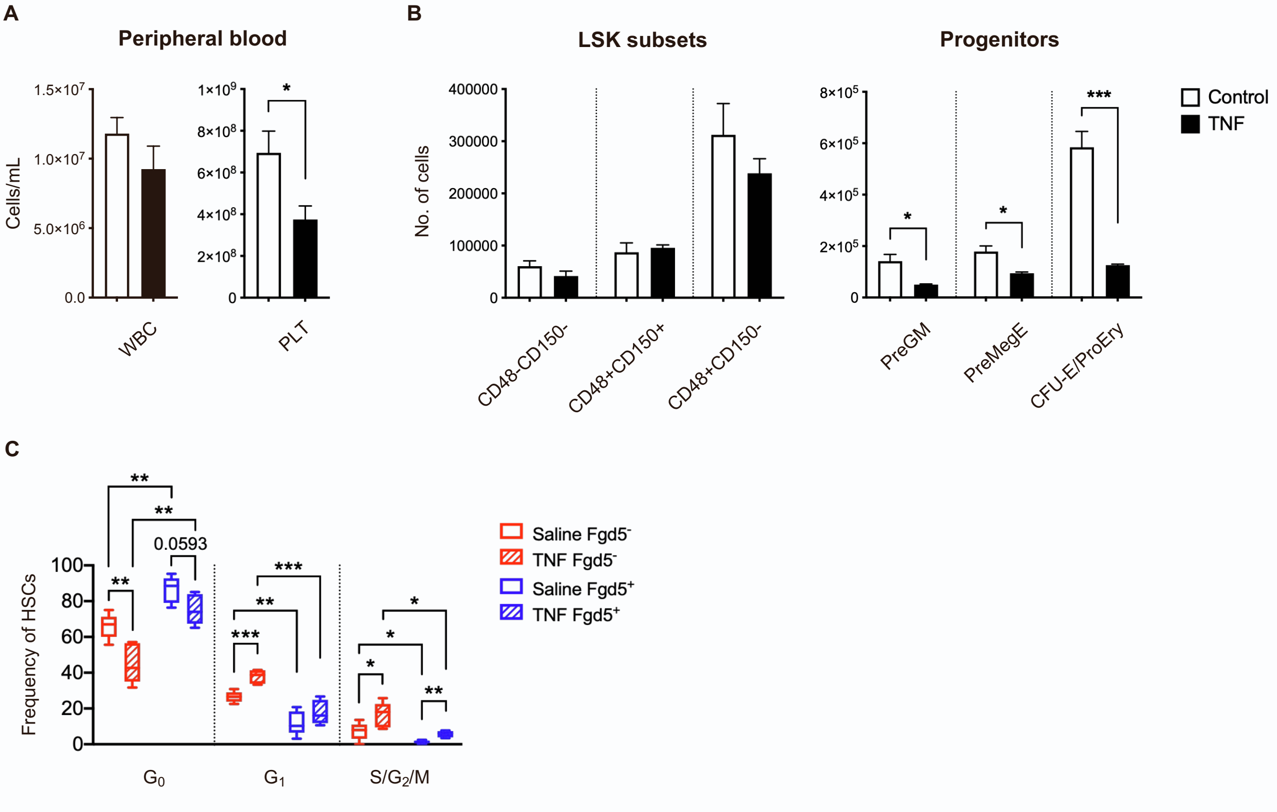

Supplementary Figure 1.

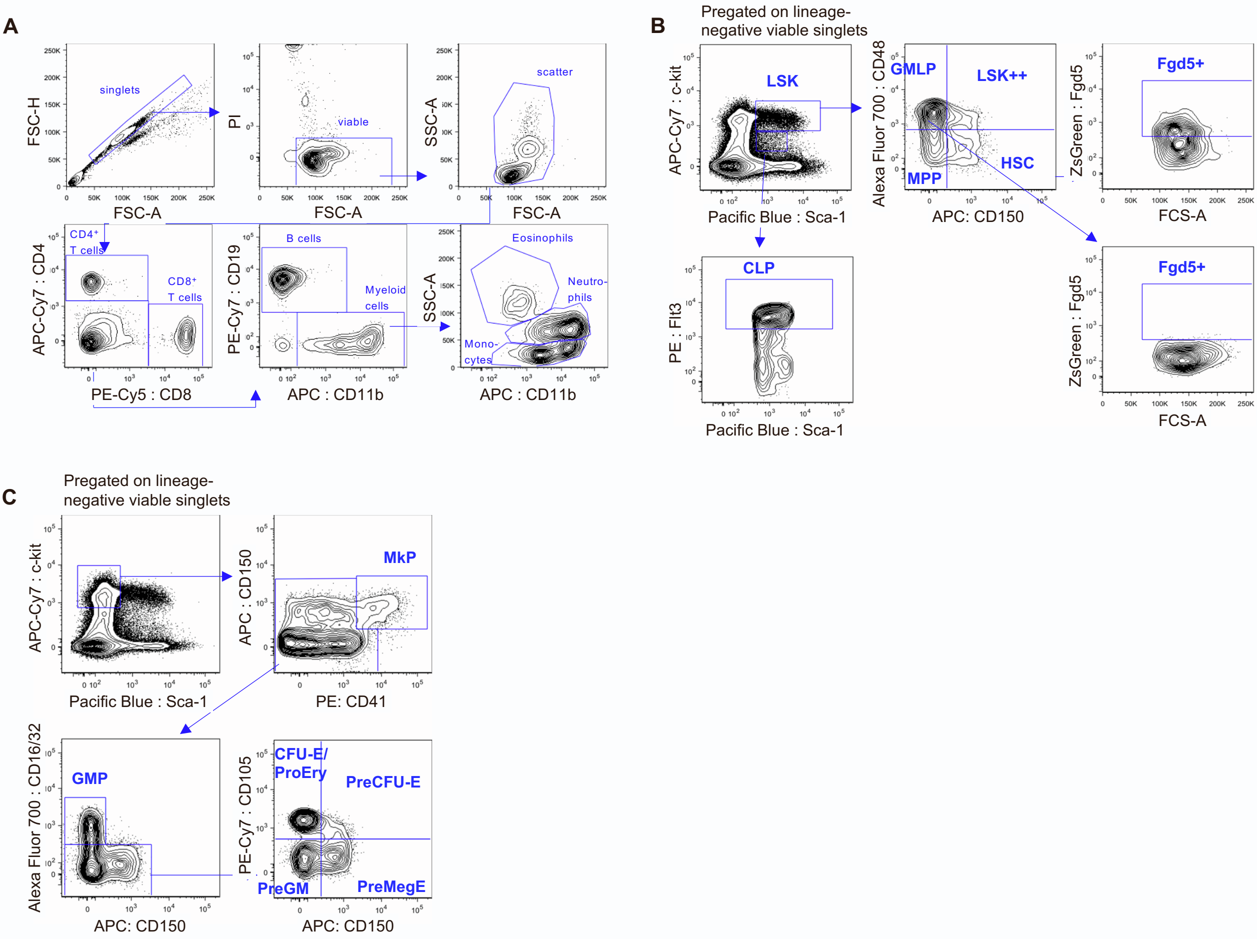

Supplementary Figure 2.

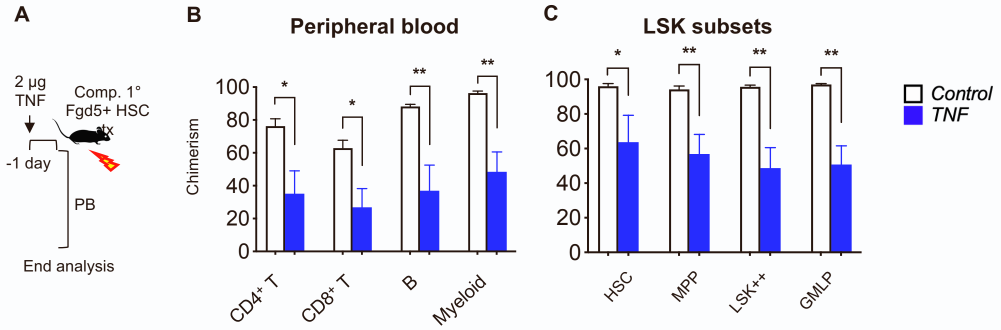

Supplementary Figure 3.

SUPPLEMENTAL INFORMATION

Supplemental figure legends

Supplementary Figure 1. [Acute TNF stimulation results in reduced PB platelet levels and BM myeloid-erythroid progenitors, and increased HSC proliferation.] Related to Figure 1. A-C depict results one day post TNF administration. **(A)** PB WBC and PLT cell concentrations. **(B)** BM cell numbers for various subsets within the LSK compartment (left) and of progenitor populations (right). TNF-treated mice n = 5, control-treated mice n = 4. Error bars represent +SEM. **(C)** Cell cycle distribution of $Fgd5^-$ and $Fgd5^+$ HSCs from saline- and TNF-treated mice. Saline n = 5, TNF n = 5. Whiskers represent min to max. Comparisons were done using unpaired two-tailed student's t-tests, *p < 0.05, **p < 0.01, ***p < 0.001, ****p < 0.0001.

Supplementary Figure 2. [Flow cytometry gating strategy for PB and BM cellular subsets.] Related to Figures 1-3. Representative flow cytometry gating for (**A**) PB populations, (**B**) BM HSC and lymphoid progenitor populations, and **(C**) BM myeloid, erythroid, and megakaryocytic progenitor populations.

Supplementary Figure 3. [Active TNF signaling reduces HSC repopulation capacity.] Related to figure 2. (**A**) Experimental strategy to assess the activity of $Fgd5^+$ HSCs receiving one dose of TNF one day prior to transplantation. Results are depicted in B-C. (**B)** PB and **(C)** BM HSPC chimerism at 20 weeks post transplantation. WT n = 6, TNF n = 5. Error bars represent +SEM. Comparisons were done using unpaired two-tailed student's t-tests, *p < 0.05, **p < 0.01, ***p < 0.001, ****p < 0.0001.

Supplementary Table 2. [Downregulated pathways in TNF-exposed HSCs identified through gene set enrichment analysis.] Related to Table 1.

Term	Overlap	P-value	Adjusted P-value	Odds Ratio	Combined Score	Genes
**Rho GTPase cycle**	11/123	1.0541317779794091E-5	0.007790033839267833	5.677541208791209	65.0658031592915	PREX1;ARHGAP10;STARD8;MCF2;RAC2;ARHGAP18;ARHGEF18;PIK3R2;SRGAP3;RHOQ;RHOBTB2
**Hemostasis pathway**	22/468	2.7818417090565628E-5	0.010278905114964	2.8970392616667353	30.38939799759139	DGKG;GUCY1A3;NFE2;SELPLG;TRPC6;DOCK8;PDGFB;PIK3R2;ITPR3;IGF1;MAPK14;GNG2;CDK5;MMRN1;INPP5D;RAC2;GNB4;SIRPA;ESAM;PRKACB;ZFPM1;SH2B1
**Lipid and lipoprotein metabolism**	22/489	5.3575924703435054E-5	0.013197536118612836	2.763740185581727	27.179756210012027	ABCA1;CERS4;HSD3B7;CDK19;MTMR3;CHD9;GBA;INPPL1;PRKAG2;PIK3R2;CROT;MED13L;SMPD2;GM2A;INPP5D;LPCAT2;ANGPTL4;HADH;PRKACB;ABCD1;PPARGC1B;ABCG1
**PI3K class IB pathway in neutrophils**	6/43	9.562137148561107E-5	0.017666048381966646	9.273028130170987	85.8229344338371	PREX1;CYTH4;INPP5D;RAC2;PRKACB;PDK1
**GATA3-mediated activation of Th2 cytokine expressio**	4/20	3.539340622779106E-4	0.048218533026207135	14.228260869565217	113.06345113427675	NFATC2;NFATC1;MAPK14;PRKACB
**G alpha (12/13) signaling events**	7/77	3.914901192926154E-4	0.048218533026207135	5.725438596491228	44.919216389146435	PREX1;GNG2;MCF2;RAC2;GNB4;ARHGEF18;PIK3R2

Supplementary Table 4. [Upregulated pathways in TNF-exposed HSCs identified through gene set enrichment analysis.] Related to Table 1.

Term	Overlap	P-value	Adjusted P-value	Odds Ratio	Combined Score	Genes
**Myc Targets V1**	35/200	1.0604112613714109E-19	5.196015180719913E-18	8.743102668475803	381.99017698961836	EIF4A1;SF3B3;ODC1;NOLC1;DDX21;GSPT1;UBE2L3;SRM;SYNCRIP;G3BP1;DHX15;USP1;EIF4H;PSMD1;SNRPB2;CCT3;CCT2;SSB;NCBP1;IFRD1;PA2G4;RAD23B;RSL1D1;PSMA4;PSMC6;TCP1;CANX;HNRNPD;MCM5;ETF1;TARDBP;ABCE1;EIF3D;KPNB1;EIF3B
**Interferon Gamma Response**	30/200	3.651428090324361E-15	5.963999214196456E-14	7.195085629188384	239.19096626997057	CD274;MVP;TNFAIP2;NLRC5;ADAR;IFIT1;ICAM1;IL18BP;SOCS3;CCL5;NAMPT;CASP4;TNFSF10;ST8SIA4;PIM1;PDE4B;STAT2;STAT3;PARP14;SOD2;NFKB1;SELP;NFKBIA;PLSCR1;ZNFX1;SERPING1;PSME2;LCP2;CD69;LY6E
**mTORC1 Signaling**	30/200	3.651428090324361E-15	5.963999214196456E-14	7.195085629188384	239.19096626997057	PSMD12;INSIG1;RPN1;USO1;ATP2A2;VLDLR;HMGCR;STIP1;SERP1;NAMPT;SSR1;ACTR3;CYB5B;XBP1;HSPA5;HMGCS1;HSPA4;EDEM1;IFRD1;M6PR;IMMT;CCT6A;SQLE;SLC7A5;PSMA4;PSMC6;PSME3;CANX;ETF1;CALR
**TNF-alpha Signaling via NF-kB**	24/200	2.635517446603224E-10	3.2285088720889497E-9	5.488636363636363	121.06159753095606	PTGER4;PFKFB3;TNFAIP2;RNF19B;TRAF1;SOD2;TANK;RELA;GEM;NFKB1;ICAM1;RHOB;NFKB2;EHD1;NFKBIA;SOCS3;CCL5;NAMPT;BCL3;PDE4B;CD69;NFKBIE;PDLIM5;BIRC3
**Unfolded Protein Response**	18/113	4.596353223270804E-10	4.5044261588053884E-9	7.563742690058479	162.62491318708126	EIF4A1;XBP1;HSPA5;EDEM1;EIF4A3;NOLC1;IFIT1;SDAD1;HERPUD1;SLC7A5;SPCS3;SERP1;DKC1;SSR1;HYOU1;CALR;ATF6;EIF4G1
**Inflammatory Response**	21/200	3.781082707334731E-8	3.08788421099003E-7	4.692008744231236	80.18957470268029	PTGER4;IL1R1;ITGB3;ATP2A2;AHR;SLC7A1;RELA;NFKB1;ICAM1;NFKBIA;IL18RAP;CCL5;NAMPT;TNFSF10;PDE4B;FFAR2;LCP2;CD69;SLC28A2;CD55;LY6E
**G2-M Checkpoint**	19/200	7.976554252623375E-7	5.583587976836363E-6	4.180497807142451	58.70083256954046	SLC12A2;HSPA8;TGFB1;CUL5;ODC1;NOLC1;SLC7A1;RAD23B;GSPT1;SQLE;SYNCRIP;SLC7A5;UCK2;DKC1;G3BP1;BCL3;HNRNPD;MCM5;KPNB1
**IL-2/STAT5 Signaling**	18/199	3.1353563363179708E-6	1.920405755994757E-5	3.9523224882340906	50.08686487338141	CYFIP1;XBP1;GPR65;ODC1;AHR;TRAF1;RHOB;SOCS2;SELP;UCK2;PLSCR1;MUC1;MAPKAPK2;TNFSF10;PIM1;ENPP1;LTB;TGM2
**IL-6/JAK/STAT3 Signaling**	11/87	1.1614789764841096E-5	6.32360776085793E-5	5.70139852674282	64.78631014850518	SOCS3;TGFB1;IL1R1;ITGB3;STAT2;STAT3;PIM1;LTB;IL2RG;IL13RA1;PF4
**Myc Targets V2**	9/58	1.3557415513554027E-5	6.643133601641474E-5	7.21595547309833	80.88059175477478	SUPV3L1;MYBBP1A;NDUFAF4;NOLC1;MPHOSPH10;MCM5;PA2G4;WDR43;SRM
**Complement**	16/200	5.0030344592495686E-5	2.0429057375269071E-4	3.44119743406985	34.07776815292405	USP14;DOCK4;HSPA5;LRP1;PREP;FN1;EHD1;PLSCR1;COL4A2;CCL5;CASP4;PIM1;SERPING1;ADAM9;LCP2;CD55
**Allograft Rejection**	16/200	5.0030344592495686E-5	2.0429057375269071E-4	3.44119743406985	34.07776815292405	SPI1;TGFB1;GPR65;IL2RG;IL27RA;ICAM1;FGR;IL18RAP;CCL5;BCL3;ST8SIA4;LCP2;LTB;ABCE1;EIF3D;PF4
**Apoptosis**	14/161	5.95897689755456E-5	2.2460759075397957E-4	3.7607385811467444	36.58456517910965	APP;PSEN2;SOD2;RELA;RHOB;DNAJA1;CASP4;TNFSF10;PMAIP1;TIMP3;CTNNB1;ETF1;CD69;BIRC3

**Androgen Response**	10/100	2.1517321329566838E-4	7.531062465348393E-4	4.364822312190733	36.85685296802441	RPS6KA3;SPCS3;STEAP4;HMGCS1;INSIG1;UAP1;PA2G4;HMGCR;PDLIM5;FKBP5
**Epithelial Mesenchymal Transition**	14/200	5.729734291110886E-4	0.0018717132017628895	2.9662058371735793	22.14175132712898	ITGB1;TGFB1;TPM4;LRP1;ITGB3;FN1;TNFRSF11B;GEM;RHOB;GLIPR1;COL4A2;COL4A1;TIMP3;TGM2
**UV Response Up**	12/158	6.81570548091752E-4	0.002046316548097335	3.2325425993985966	23.568826236413006	SULT1A1;NFKBIA;CYB5B;DNAJA1;STIP1;NXF1;RPN1;DDX21;SOD2;HSPA13;ICAM1;RHOB
**Protein Secretion**	9/96	7.099465575031571E-4	0.002046316548097335	4.056217345872518	29.40887724311624	RPS6KA3;COPB2;TMED10;SNAP23;USO1;M6PR;ANP32E;TMED2;ATP1A1
**Coagulation**	11/138	7.56346569193817E-4	0.0020589434383609466	3.402900448802926	24.45668248774843	ARF4;LRP1;GDA;ITGB3;PREP;FN1;ADAM9;SERPING1;TIMP3;ISCU;PF4
**Cholesterol Homeostasis**	7/74	0.002597997750445961	0.0067000994616764255	4.084296825730503	24.31387710928505	FDPS;SQLE;PLSCR1;HMGCS1;CTNNB1;HMGCR;HSD17B7
**Apical Junction**	11/200	0.012826455296009972	0.03142481547522443	2.279285660624403	9.92912772222282	ITGB1;CD274;INSIG1;NF1;ADAM9;MSN;TNFRSF11B;MADCAM1;PIK3CB;TRAF1;ICAM1
**Fatty Acid Metabolism**	9/158	0.018902871112150863	0.04410669926168535	2.360829774252593	9.368814750853424	HSP90AA1;HMGCS1;ODC1;DLST;SERINC1;HSD17B7;GLUL;PRDX6;DLD

SUPPLEMENTAL INFORMATION

Supplemental table legends

Supplementary Table 2. [Downregulated pathways in TNF-exposed HSCs identified through gene set enrichment analysis.] Related to Table 1. BioPlanet gene sets, adj. p-value < 0.05.

Supplementary Table 4. [Upregulated pathways in TNF-exposed HSCs identified through gene set enrichment analysis.] Related to Table 1. MSigDB_Hallmark gene sets, adj. p-value < 0.05.
